# Kindness Rewarded

**Published:** 1868-09

**Authors:** 


### KINDNESS REWARDED.

A young man was left severely wounded on the plains of Chalmette, when Jackson vanquished Packenham on the "8th of January," forever memorable; he was taken to a place of safety and tenderly cared for, by a poor soldier. Just fifty years later, Judah Touro bequeathed to his old friend, Shepherd, fifty thousand dollars, made him an executor of an estate of millions, and constituted him residuary legatee, amounting to a large sum.

Captain Robert H. Pearson of San Francisco, has bequeathed one thousand dollars to the wife of Capt. William Stout, of Stamford, Connecticut, for kindness to him during an illness in New York City in 1859; and to Mrs. Eliza Jane Winsor, for her care of him during a severe illness in 1867, two thousand dollars in gold. The grateful Captain desired to be buried by the side of his mother in Greenwood Cemetery, near New-

York, lot 5.668. No doubt the women above named were so full of a woman's noble ioving nature, that they could not have helped doing these good deeds, and they had pleasure in its performance ; but it is quite as sweet to know that kindness is appreciated ; may they not fail to find a double portion of it meeted out to them by loving hearts, when they come to close their eyes on all things earthly, to open them again on the face of him who graciously said in the days of his flesh, "Inasmuch as ye did it unto one of the least of these my brethren, ye have done it unto me."

